# A comparison of self-reported and proxy-reported health utilities in children: a systematic review and meta-analysis

**DOI:** 10.1186/s12955-021-01677-0

**Published:** 2021-02-05

**Authors:** Mingyu Jiang, Yue Ma, Minghui Li, Rui Meng, Aixia Ma, Pingyu Chen

**Affiliations:** 1 Department of Health Economics, China Pharmaceutical University, Nanjing, China; 2 Department of Clinical Pharmacy and Translational Science, University of Tennessee Health Science Center, Memphis, USA; 3 Center for Pharmacoeconomics and Outcomes Research, China Pharmaceutical University, Nanjing, China

**Keywords:** Health utility, Quality of life, Children, Proxy, Meta-analysis

## Abstract

**Objective:**

This study aimed to conduct a systematic review and meta-analysis to compare differences in health utilities (HUs) assessed by self and proxy respondents in children, as well as to evaluate the effects of health conditions, valuation methods, and proxy types on the differences.

**Methods:**

Eligible studies published in PubMed, Embase, Web of Science, and Cochrane Library up to December 2019 were identified according to PRISMA guidelines. Meta-analyses were performed to calculate the weighted mean differences (WMDs) in HUs between proxy- versus self-reports. Mixed-effects meta-regressions were applied to explore differences in WMDs among each health condition, valuation method and proxy type.

**Results:**

A total of 30 studies were finally included, comprising 211 pairs of HUs assessed by 15,294 children and 16,103 proxies. This study identified 34 health conditions, 10 valuation methods, and 3 proxy types. In general, proxy-reported HUs were significantly different from those assessed by children themselves, while the direction and magnitude of these differences were inconsistent regarding health conditions, valuation methods, and proxy types. Meta-regression demonstrated that WMDs were significantly different in patients with ear diseases relative to the general population; in those measured by EQ-5D, Health utility index 2 (HUI2), and Pediatric asthma health outcome measure relative to Visual analogue scale method; while were not significantly different in individuals adopting clinician-proxy and caregiver-proxy relative to parent-proxy.

**Conclusion:**

Divergence existed in HUs between self and proxy-reports. Our findings highlight the importance of selecting appropriate self and/or proxy-reported HUs in health-related quality of life measurement and economic evaluations.

## Introduction

Economic evaluation is used to compare the costs and consequences of alternative healthcare interventions. The measurements of various costs are similar; but the forms of health outcomes vary by different evaluation techniques, including cost-effectiveness analysis (CEA), cost–benefit analysis (CBA), and cost-utility analysis (CUA) [1]. The CUA has been recommended by many government agencies to support the decision-making process and increasingly used in economic evaluations [2–4]. In CUA, quality-adjusted life years (QALYs), which combine health utilities (HUs) and length of life into a single metric, are used as health outcomes. The HU which can reflect the societal preference for different health states is usually a value between 0 and 1, where 0 represents death and 1 represents perfect heath. There are also HUs for coditions worse than death, which theoretically has no minimum limit [5]. However, in order to avoid the large impact that a negative value has on the average HU calculation, it is usually converted to a value between − 1 and 0, which is symmetrical to the value range for health conditions better than death [1].

When estimating HUs associated with different health states, both direct and indirect valuation methods can be used [5]. Direct methods indicate that the assessment and measurement process can be completed in one step. Common direct methods include the visual analogue scale (VAS), standard gamble (SG), and time trade-off (TTO) techniques [1]. Indirect methods rely on the multi-attribute utility instruments (MAUIs) which comprise two main elements: a descriptive system for measuring health states and a scoring algorithm for valuing the health states defined by the descriptive system [6]. Commonly used indirect methods for children include the Health Utilities Index (HUI) [7], the EuroQol 5-dimension (EQ-5D) Youth version (EQ-5D-Y) [8], and the Child Health Utility 9-Dimension (CHU9D) [9].

It is documented in the literature that there are more challenges when measuring HUs for children than for adults [10]. The cognitive capability and the reading level of children are lower than that of adults, which will make it very challenging applying valuation methods to obtain their HUs [11]. Therefore, proxy-assessment is needed on this occasion. Proxy-assessment is a way to measure children’s HUs by children’s proxies such as their parents, clinician or other people knowing children’s health states instead of themselves. However, the consistency between proxy-reported and self-reported HU values is controversial. Mark et al*.* [12] found that the interrater agreement was the highest between cancer survivors and parents and the lowest between controls and physicians or teachers. Miguel et al*.* [13] found that the level of agreement was poor in the HRQoL assessment between children with cerebral palsy and parents in all the questionnaire domains of EQ-5D-Y and fair or poor in the visual analogue scale. Bull et al*.* [14] found that parents of children with mild to moderate health conditions rated utilities similar to their children.

Reviews involving self-reported and proxy-reported HUs have been conducted in children. Khadka et al*.* [15] used narrative synthesis and found that utilities derived from children or proxies are not interchangeable. Kwon et al*.* [11, 16] identified proportions of respondent types and analyzed the influence of proxy type on HUs obtained by HUI3 and VAS. However, there is no systematic review using quantitative analysis to compare the differences between self-reported and proxy-reported HU values, and exploring the potential influence factors. Since Proxy-assessment are commomly used for obtaining children’s HUs due to children’s limited cognitive capability regardless of the severity of children’s health condition, it is of necessity and importance to understand the differences of magnitude and direction between self and proxy-reported HUs, as well as which factors have potential impact on the differences [11]. Based on this, the researchers may select proper methods or proxies to measure the primary HUs, or to select the proper HUs for performing economic evaluations. Therefore, we conducted this study to compare the differences between self and proxy-reported HUs and to explore the effects of different health conditions, valuation methods, and proxy types on these differences.

## Methods

### Systematic review

The systematic review was performed according to the Preferred Reporting Items for Systematic Reviews and Meta-Analyses (PRISMA) guidelines [17]. The full PRISMA checklist was included in Additional file 1: Appendix 1. The following databases were searched for studies between inception and December 31, 2019: Pubmed, Embase, Web of Science, and Cochrane Library. The search terms and the detailed search strategy was outlined in Additional file 1: Appendix 2.

Studies were included if they (1) reported HUs assessed by pairwise (children and proxies) populations, (2) contained participants were aged under 18 years, and (3) were published in English. Studies were excluded if they (1) did not focus on pediatric populations, (2) did not include HU values, (3) proxy respondents reported their own HUs, (4) were published in the form of systematic review, meta-analysis, abstract, or dissertation. The literature search and screening were conducted independently by two investigators. Any disagreements were adjudicated by senior investigators.

### Data extraction

The following information was extracted: general characteristics of studies and participants, study designs, health conditions, valuation methods, and types of proxy. Data extracted or calculated from studies included sample size and mean, as well as standard deviation (SD) of HUs. The VAS scores were divided by 100 if the primary data were assessed using a 0–100 scale. In addition, health conditions were classified according to the International Classification of Diseases 10 revision (ICD-10) chapters [16]. Two investigators independently extracted the data from the full text. If any inconsistency was found, senior investigators reviewed the original literature and made a final decision.

### Quality assessment

The risk of bias was evaluated using the Newcastle–Ottawa Scale for longitudinal studies [18] and an adapted form for cross-sectional and patient case series studies [19]. Specific assessment results were listed in Additional file 1: Appendix 3. Two investigators independently conduct the quality assessment and inconsistent results were resolved by senior investigators.

### Statistical analysis

Weighted mean difference (WMD) is a summary statistic commonly used for meta analysis of continuous data, it can be used to measure the absolute difference between the mean value in two groups [20]. Therefore, we conducted meta-analysis to estimate WMD which was defined as proxy-reported HUs minus self-reported HUs, and associated 95% confidence intervals (CI), by specific health conditions, valuation methods, and proxy types. The mean and SD of HU values as well as sample sizes were included to pool the WMDs. The heterogeneity was quantified using the I^2^ statistic, fixed effects models were applied if the value of I^2^ was smaller than 50%; otherwise, random effects models were used [21, 22].

Meta-regression can be used to investigate differences for categorical independent variables [23].In Meta-regressions, the dependent variable is the effect estimate, the independent variables are characteristics of studies that might influence the size of effect. The regression coefficients will estimate how the effect in each subgroup differs from a reference subgroup [20]. Therefore, we performed meta-regression using linear mixed-effects models with restricted maximum likelihood method to evaluate the effects of different factors on the difference in HUs between self-reports and proxy-reports, after controlling for methodological factors and study specific random effects not accounted for by the independent variables [20]. The dependent variable were WMDs estimated from the meta-analysis, and the independent variables included health conditions, valuation methods, and proxy types. Independent variables regarding health condition were introduced on condition level instead of diagnosis level since too many diagnosis types existed. The coefficients and 95% CIs were reported and *P* values < 0.05 were deemed to be statistically significant. *P* values were also adjusted by using a Monte Carlo permutation test with 1000 iterations to obtain more realistic values. Statistical analyses were performed using Stata software, version 15.0.

## Results

### Systematic review

**Fig. 1:**
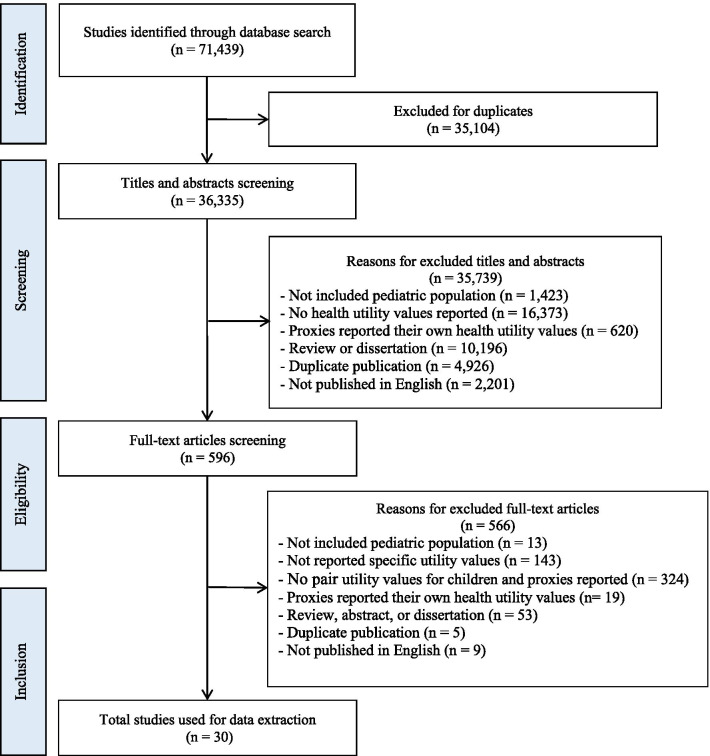
Flow diagram of literature search and study identification

Figure 1 presented the flow chart of the literature search and screening. The literature search identified 71,439 articles and 35,104 articles were excluded due to duplicates. A total of 35,739 articles were excluded by titles and abstracts screening, the main reason for exclusion was no HU values reported. Hence, 596 articles entered full-text articles screening and 566 articles were excluded mainly for not reporting pairwise HU values. Finally, 30 articles comprising 211 pairs of self-reported and proxy-reported HUs were included in meta-analyses [9, 24–52].

### Study characteristics

Table 1 summarized the characteristics of the included studies. These studies were published between 1994 and 2019, and most of them were conducted in USA (23%), UK (17%), and Canada (10%). In terms of study design, 15 studies were cross-sectional (50%), 7 were longitudinal (23%) and 8 were patient case series (27%). There were 211 pairs of HUs assessed by 15,294 children and 16,103 proxies, covering the general population and individuals with different health conditions.

**Table 1:** Characteristics of included studies

Author (year)	Country	Study design	Health conditions	Age (years)	Gender (% of male)	Sample size	Valuation method		Proxy type
							Direct	Indirect	
Cardarelli (2006)	Italy	CS	Brain tumour/mixed cancer diagnosis/leukaemia/lymphoma	R: 12–28	NA	184		HUI2	Parents
Fu (2006)	4 countries	CS	Mixed cancer diagnosis	R: 3.4–25.8; Median: 12.8	56.0%	680		HUI2; HUI3	Parents; clinicians
Glaser (1999)	UK	CS	Brain tumour	R: 10–16	NA	153		HUI2; HUI3	Parents; clinicians
Penn (2011)	UK	L	Brain tumour/general	Brain tumour: R: 3.6–16.4; Median: 11.1 General: R: 5.1–18.9; Median: 10.7	NA	43		HUI3	Parents
Verrips (2001)	Netherlands	C	VLBW/VP	M: 14.3	NA	406		HUI3	Parents
Wolke (2013)	Germany	C	VLBW/VP/general	M: 13	VLBW/VPT: 55.2% General: 49.5%	542		HUI3	Parents
Saigal (1999)	Canada	C	ELBW/EP	M(SD): 14.2(1.6)	NA	1,056	SG		Parents
Lee (2011)	USA	C	Diabetes	HUI3: R:8–18; M(SD): 13.7(3.1) TTO: R: 15–18	NA	326	TTO	HUI3	Parents
Belfort (2011)	USA	CS	Overweight or obese/general	Overweight or obese: R:8–17; 12–17 General: R:8–18; 8–18	NA	304		HUI3	Parents
Brunner (2003)	Canada	CS	MSKD	R: 8–18	NA	202	SG; VAS	HUI3	Parents
Brunner (2004)	USA	L	MSKD	R: 3–18	NA	410	SG; VAS		Parents
Czyzewski (1994)	USA	CS	Cystic fibrosis	R: 0.2–17.9; M(SD): 8.74(4.86)	NA	55		QWB	Parents
Gerald (2012)	USA	CS	Asthma	R: 6–12	63.6%	2,806		PAHOM	Parents
Kulpeng (2013)	Thailand	C	Bacteremia/epilepsy/hearing loss/lung disease/meningitis/otitis media	R: 5–14	NA	296	EQ-5D; VAS	EQ-5D; HUI3; HUI2	Caregivers
Trent (2011)	USA	C	Pelvic inflammatory disease	R: 12–19	0%	1,340	TTO; VAS		Parents
Sung (2004)	Canada	CS	Asthma/Chronic illness/Injury/Stroke	R: 12–18; M(SD): 13.7(1.7)	55.0%	235	SG; TTO; VAS	HUI2; HUI3	Parents
Jelsma-Ramma (2010)	South Africa	C	Chronic illness/general	R: 7–12	Chronic illness: 74.0% General: 45.0%	628	EQ-5D-Y; VAS		Parents
Vermeulen (2017)	Netherlands	C	ODD/CD/DBD/ODD + ADHD/CD + ADHD/DBD + ADHD/ODD + SA/CD + SA	R: 4–12; M:9; R:12–18; M:15	NA	688	EQ-5D-3L; VAS		Parents
Baumann (2016)	Germen	L	VLBW/VPT/General	VLBW/VPT: M:13; 26 General: M:13; 26	VLBW/VPT: 45.3% General: 53.7%	792		HUI3	Parents
Robertson (2016)	England	L	Overweight or obese	R: 6–11; M(SD): 9.43(1.61)	42.9%	626	VAS	EQ-5D-Y	Parents
Hanberger (2009)	Sweden	C	Diabetes	R: 2.6–19.6; M(SD): 13.2(3.9)	52.3%	148		EQ-5D	Parents
Rhodes (2012)	USA	C	Diabetes	R: 12–18; M(SD): 15.5(2)	32.9%	107		HUI3	Parents
Sims-williams (2017)	Uganda	C	Spina bifida	R: 10–14	56.0%	125	VAS	HUI3	Caregivers
Medeiros (2019)	North America	C	Medulloblastoma	R: 0.33–14.95; 1–17; M(SD): 6.82(3.41); 6.48(3.9)	68.0%; 76.0%	152		HUI2; HUI3	Parents
Kirkham (2019)	4 countries	C	Prolonged acute convulsive seizures	R: 13–16; M(SD): 8.8(3.8)	54.9%	54		EQ-5D-3L	Parents; clinicians
Lopez-Bastida (2019)	Spain	C	Diabetes	M(SD): 10.5(3.9); 11.7(3.9); 10.8(4); 11.9(3.4); 11(3.9)	52.8%; 53.7%; 56%; 43.1%; 53.2%	801	EQ-5D-Y VAS		Caregivers
Perez-Sousa (2018)	Spain	L	Overweight or obese	R: 6–14; 6–13; M(SD): 9.6(2.1); 8.7(1.6)	55.0%; 47.0%	302	EQ-5D-Y VAS		Parents
Bray (2017)	UK	C	Paediatric mobility impairment	R: 6–15; 16–18; 6–18	38.5%	100	EQ-5D VAS	EQ-5D-Y; HUI2; HUI3	Parents
Creswell (2017)	UK	L	Anxiety disorders	M(SD): 9.28(1.83)	47.0%	339		CHU-9D	Parents
Shi (2017)	Japan	L	General	M: 11.46	50.0%	1,308	EQ-5D VAS		Parents

*CS* case series, *L* longitudinal, *C* cross-sectional, *VLBW/VP* very low birth weight/very preterm, *ELBW/EP* extremely low birth weight/extremely preterm, *ODD* oppositional defiant disorder, *CD* conduct disorder, *DBD* disruptive behaviour disorder, *ADHD* attention deficit hyperactivity disorder, *SA* substance abuse, *R* range, *M* mean, *SD* standard deviation, *VAS* visual analogue scale, *SG* standard gamble, *TTO* time trade-off, *HUI* health utility index, *EQ-5D-Y* EuroQol 5-dimension youth version, *CHU9D* child health utility 9-dimension, *QWB* quality of well-being scale, *PAHOM* pediatric asthma health outcome measure

Nine valuation instruments were identified. Direct instruments contained VAS (74 pairs; 35%), SG (15 pairs; 7%), and TTO (7 pairs, 3%); while indirect instruments contained EQ-5D (10 pairs, 5%), EQ-5D-Y (9 pairs, 4%), HUI2 (22 pairs, 10%), HUI3(37 pairs, 15%), CHU-9D (6 pairs, 3%), QWB (1 pair, 0.5%), and Pediatric asthma health outcome measure (PAHOM) (30 pairs, 14%). The most commonly used proxy respondent type was parents (169 pairs, 80%), followed by caregivers (35 pairs, 17%) and clinicians (7 pairs, 3%).

Additional file 1: Appendix 3 presented the quality of included studies. In general, all the studies showed low risks of bias, which indicated high quality.

### WMDs in HUs by health conditions

We pooled the WMDs of HUs between self-reports and proxy-reports by health conditions (Table 2). On this basis, the WMDs assessed by different valuation methods (Additional file 1: Appendix 4) and proxy types (Additional file 1: Appendix 5) were estimated, respectively. In Table 2, the proxy-reported HUs were significantly higher than self-reported ones in general (WMD: 0.034; 95% CI: 0.021, 0.047), overweight or obese (WMD: 0.059; 95% CI: 0.045, 0.073), cystic fibrosis (WMD: 0.030; 95% CI: 0.005, 0.055), and ELBW/EP (WMD: 0.065; 95% CI: 0.023, 0.106) populations. While the proxy-reported HUs were significantly lower in meningitis (WMD: − 0.117; 95% CI − 0.167, − 0.067), pediatric mobility impairment (WMD: − 0.059; 95% CI − 0.059, − 0.027), prolonged acute convulsive seizures (WMD: − 0.265; 95% CI − 0.344, − 0.186), asthma (WMD: − 0.034; 95% CI: − 0.039, − 0.029), lung disease (WMD: − 0.030; 95% CI − 0.057, − 0.003), spina bifida (WMD: − 0.044; 95% CI − 0.078, − 0.010), injury (WMD: − 0.083; 95% CI − 0.155, − 0.012), and chronic illness populations (WMD: − 0.047; 95% CI − 0.080, − 0.013).

**Table 2:** Weighted mean differences in health utilities between self- and proxy-reports by health conditions

Health conditions*	N groups (N participants)	N groups (N proxies)	WMD† (95% CI)	*P*	I^2^
General populations	9 (2746)	9 (2746)	0.034 (0.021, 0.047)	< 0.001	75.5%
Infectious and parasitic diseases					
Bacteremia	4 (36)	4 (28)	0.005 (− 0.044, 0.055)	0.829	38.4%
Meningitis	4 (28)	4 (48)	− 0.117 (− 0.167, − 0.067)	< 0.001	49.9%
Cancer					
Brain tumour	8 (194)	8 (232)	0.055 (− 0.010, 0.119)	0.099	62.0%
Leukaemia/lymphoma	1 (33)	1 (79)	− 0.040 (− 0.108, 0.028)	0.251	–
Medulloblastoma	4 (152)	4 (152)	− 0.013 (− 0.059, 0.034)	0.589	0.0%
Mixed cancer diagnosis	6 (611)	6 (991)	0.053 (− 0.026, 0.132)	0.188	95.7%
Endocrine, nutritional and metabolic disorders					
Diabetes	9 (1382)	9 (1503)	0.004 (− 0.005, 0.014)	0.351	0.0%
Overweight or obese	18 (1080)	18 (1080)	0.059 (0.045, 0.073)	< 0.001	94.9%
Cystic fibrosis	1 (55)	1 (199)	0.030 (0.005, 0.055)	0.017	/
Mental and behavioral disorders‡					
Anxiety disorders	6 (339)	6 (310)	0.011 (− 0.001, 0.024)	0.077	0.0%
CD	3 (86)	3 (46)	0.085 (− 0.113, 0.282)	0.400	94.2%
CD + SA	3 (86)	3 (46)	0.085 (− 0.043, 0.213)	0.193	84.1%
CD + ADHD	3 (86)	3 (46)	0.075 (− 0.096, 0.247)	0.390	91.8%
DBD	3 (86)	3 (46)	0.072 (− 0.110, 0.254)	0.438	93.3%
DBD + SA	3 (86)	3 (46)	0.055 (− 0.068, 0.177)	0.382	84.2%
DBD + ADHD	3 (86)	3 (46)	0.052 (− 0.072, 0.176)	0.412	83.9%
ODD	3 (86)	3 (46)	0.101 (− 0.045, 0.246)	0.174	90.5%
ODD + SA	3 (86)	3 (46)	0.121 (− 0.050, 0.291)	0.167	89.0%
ODD + ADHD	3 (86)	3 (46)	0.091 (− 0.127, 0.310)	0.414	95.0%
Paediatric mobility impairment	12 (100)	12 (100)	− 0.059 (− 0.091, − 0.027)	< 0.001	7.6%
Prolonged acute convulsive seizures	2 (54)	2 (556)	− 0.265 (− 0.344, − 0.186)	< 0.001	0.0%
Nervous system disorders					
Epilepsy	4 (64)	4 (16)	− 0.009 (− 0.040, 0.022)	0.560	0.0%
Diseases of the ear					
Hearing loss	4 (60)	4 (28)	0.068 (− 0.005, 0.141)	0.067	90.0%
Otitis media	4 (28)	4 (44)	0.063 (− 0.039, 0.165)	0.225	86.8%
Circulatory system disorders					
Stroke	2 (44)	2 (44)	− 0.006 (− 0.088, 0.077)	0.893	0.0%
Respiratory system disorders					
Asthma	32 (2850)	32 (2850)	− 0.034 (− 0.039, − 0.029)	< 0.001	34.6%
Lung disease	8 (80)	8 (80)	− 0.030 (− 0.057, − 0.003)	0.031	0.7%
Musculoskeletal disorders					
MSKD	16 (612)	16 (900)	0.019 (− 0.001, 0.039)	0.068	43.6%
Genitourinary system disorders					
Pelvic inflammatory disease	10 (1340)	10 (1210)	0.123 (0.101, 0.144)	< 0.001	25.5%
Conditions originating in the perinatal period					
ELBW/EP	4 (1056)	4 (1100)	0.065 (0.023, 0.106)	0.002	45.8%
VLBW/VP	6 (1053)	6 (1053)	0.009 (− 0.016, 0.034)	0.481	98.9%
Congenital malformations					
Spina bifida	2 (125)	2 (132)	− 0.044 (− 0.078, − 0.010)	0.012	93.2%
Injury, poisoning and other consequences of external causes					
Injury	2 (44)	2 (44)	− 0.083 (− 0.155, − 0.012)	0.023	0.0%
Chronic illness					
Chronic illness	6 (164)	6 (164)	− 0.047 (− 0.080, − 0.013)	0.007	45.8%

*ODD* oppositional defiant disorder, *CD* conduct disorder, *DBD* disruptive behaviour disorder, *ADHD* attention deficit hyperactivity disorder, *SA* substance abuse, *VLBW/VP* very low birth weight/very preterm, *ELBW/EP* extremely low birth weight/extremely preterm

*Health conditions were reported based on ICD-10 categories

^†^Proxy-reported health utilities minus self-reported health utilities

^‡^Plus indicates patients have multiple diagnoses

### WMDs in HUs by valuation methods

In Additional file 1: Appendix 4, VAS scores assessed by proxies were higher than those assessed by children themselves in general (WMD: 0.041; 95% CI 0.019, 0.064), overweight or obese (WMD: 0.058; 95% CI 0.037, 0.079) and pelvic inflammatory disease (WMD: 0.146; 95% CI 0.119, 0.714) populations, while lower in patients with spina bifida (WMD: − 0.061; 95% CI − 0.072, − 0.050). Proxy-reported HUs assessed by TTO were higher in pelvic inflammatory disease patients (WMD: 0.085; 95% CI 0.051, 0.120) and lower in chronic illness patients (WMD: − 0.150; 95% CI − 0.287, − 0.013). Proxy-reported HUs assessed by SG were higher in ELBW/EP (WMD: 0.065; 95% CI: 0.023, 0.106) populations.

For HUI2, proxy-reported HU values were higher for hearing loss patients (WMD: 0.080; 95% CI 0.033, 0.127) and otitis media patients (WMD: 0.120; 95% CI 0.052, 0.188), and lower for patients with meningitis (WMD: − 0.140; 95% CI − 0.216, − 0.064), paediatric mobility impairment (WMD: − 0.043; 95% CI − 0.087, 0.000) and chronic illness (WMD: − 0.130; 95% CI − 0.234, − 0.026). For HUI3, proxies generated higher HUs in general (WMD: 0.029; 95% CI 0.016, 0.042), mixed cancer diagnosis (WMD: 0.186; 95% CI 0.137, 0.235), diabetes (WMD: 0.020; 95% CI 0.003, 0.037), overweight or obese (WMD: 0.093; 95% CI 0.084, 0.103), hearing loss (WMD: 0.170; 95% CI: 0.096, 0.244) and otitis media (WMD: 0.200; 95% CI 0.097, 0.303) populations; and generated lower HUs in meningitis (WMD: − 0.160; 95% CI − 0.304, − 0.016), paediatric mobility impairment (WMD: − 0.060; 95% CI − 0.112, − 0.008) and spina bifida (WMD: − 0.026; 95% CI − 0.040, − 0.012) patients.

Regarding HUs obtained by EQ-5D, proxies reported lower values for meningitis patients (WMD: − 0.230; 95% CI − 0.383, − 0.077) and patients with prolonged acute convulsive seizures (WMD: − 0.265; 95% CI − 0.344, − 0.186), and higher values in hearing loss population (WMD: 0.070; 95% CI 0.032, 0.108). In addition, children-specific instrument, EQ-5D-Y, was used in overweight or obese and paediatric mobility impairment populations, CHU-9D was used in anxiety disorders population, and disease-specific instrument, PAHOM, was used for asthma patients. All the P-values were less than 0.05 except for HUs assessed by CHU-9D.

### WMDs in HUs by proxy types

In Additional file 1: Appendix 5, parent-reported HUs were statistically significantly higher than self-reported HUs in general (WMD: 0.034; 95% CI 0.021, 0.047), MSKD (WMD: 0.019; 95% CI 0.005, 0.033), pelvic inflammatory disease (WMD: 0.121; 95% CI 0.096, 0.146), and ELBW/EP (WMD: 0.075; 95% CI 0.048, 0.103) populations. On the contrary, parents reported lower HUs in patients diagnosed with paediatric mobility impairment (WMD: − 0.058; 95% CI − 0.087, − 0.028), prolonged acute convulsive seizures (WMD: − 0.270; 95% CI − 0.382, − 0.158), asthma (WMD: − 0.034; 95% CI − 0.039, − 0.029), injury (WMD: − 0.083; 95% CI − 0.155, − 0.012) and chronic illness (WMD: − 0.047; 95% CI − 0.080, − 0.013).

HUs reported by clinicians were higher in brain tumour patients (WMD: 0.104; 95% CI 0.054, 0.155) and mixed cancer diagnosis patients (WMD: 0.186; 95% CI 0.137, 0.235), while lower in patients with prolonged acute convulsive seizures (WMD: − 0.260; 95% CI − 0.373, − 0.147). In addition, caregiver-reported HUs were statistically significantly lower than self-reported ones in meningitis (WMD: − 0.117; 95% CI − 0.167, − 0.067), lung disease(WMD: − 0.030; 95% CI − 0.057, − 0.003), and spina bifida (WMD: − 0.044; 95% CI − 0.078, − 0.010) populations.

### Differences in WMDs

The results of the meta-regression were shown in Table 3. The general population, VAS instrument, and proxy assessment by parents were set as the reference. The constant indicated that parent-reported HUs were higher than self-reported HUs in the general population (β: 0.047; 95% CI 0.004, 0.090) when the VAS instrument was applied. We determined statistical significance based on the adjusted *P* values which estimated by Monte Carlo permutation test. Compared with the general population applying the VAS instrument and parental proxy, the HU values of WMDs between children and their proxies were significantly higher in patients diagnosed with diseases of the ear (β: 0.127; 95% CI 0.046, 0.209); and were significantly lower in patients applying EQ-5D (β: − 0.110; 95% CI − 0.163, − 0.058), HUI2 (β: − 0.067; 95% CI − 0.111, − 0.024), and PAHOM (β: − 0.104; 95% CI − 0.175, − 0.034).

**Table 3:** Meta-regression of weighted mean differences in health utilities between self- and proxy-reports

	β	95% CI	*P*	Adjusted *P* †
Constant	0.047	(0.004,0.090)	0.034	
Health condition*				
General population	Reference			
Infectious and parasitic diseases	0.000	(− 0.088, 0.088)	0.998	1.000
Cancer	0.014	(− 0.049,0.077)	0.655	1.000
Endocrine, nutritional and metabolic disorders	0.024	(− 0.024, 0.073)	0.330	0.997
Mental and behavioral disorders	0.004	(− 0.044, 0.052)	0.876	1.000
Nervous system disorders	0.054	(0.040, 0.148)	0.256	0.986
Diseases of the ear	0.127	(0.046, 0.209)	0.002	0.024
Circulatory system disorders	− 0.050	(− 0.181, 0.082)	0.459	1.000
Respiratory system disorders	0.021	(− 0.054, 0.096)	0.583	1.000
Musculoskeletal disorders	− 0.030	(− 0.086, 0.026)	0.288	0.994
Genitourinary system disorders	0.098	(0.031, 0.166)	0.005	0.055
Conditions originating in the perinatal period	− 0.010	(− 0.066, 0.045)	0.711	1.000
Congenital malformations	− 0.020	(− 0.121, 0.082)	0.703	1.000
Injury, poisoning and other consequences of external causes	− 0.127	(− 0.255, 0.002)	0.053	0.490
Chronic illness	− 0.095	(− 0.170, − 0.020)	0.013	0.154
Valuation methods				
VAS	Reference			
SG	0.007	(− 0.040, 0.054)	0.767	1.000
TTO	− 0.059	(− 0.127, 0.008)	0.083	0.658
EQ-5D	− 0.110	(− 0.163, − 0.058)	< 0.001	0.001
EQ-5D-Y	− 0.045	(− 0.095, 0.006)	0.085	0.672
HUI2	− 0.067	(− 0.111, − 0.024)	0.002	0.025
HUI3	− 0.020	(− 0.052, 0.013)	0.228	0.967
CHU-9D	− 0.041	(− 0.094, 0.012)	0.132	0.837
QWB	− 0.041	(− 0.158, 0.076)	0.489	1.000
PAHOM	− 0.104	(− 0.175, − 0.034)	0.004	0.048
Proxy types				
Proxy assessment by parents	Reference			
Proxy assessment by clinicians	0.083	(0.016, 0.151)	0.016	0.185
Proxy assessment by caregivers	− 0.061	(− 0.114, − 0.008)	0.025	0.274

*VAS* visual analogue scale, *SG* standard gamble, *TTO* time trade-off, *HUI* health utility index, *EQ-5D-Y* EuroQol 5-dimension youth version, *CHU9D* child health utility 9-dimension, *QWB* quality of well-being scale, *PAHOM* pediatric asthma health outcome measure

*Health conditions were reported based on ICD-10 categories

^†^*P* values were adjusted by Monte Carlo permutation test with 1000 iterations

### Main findings

According to the results from WMDs of different health conditions, we found that for mild health conditions such as the general population, overweight or obese, and ELBW/EP, proxies tended to report higher HUs than children themselves; however, proxies tended to report lower HUs for severe diseases such as meningitis, pediatric mobility impairment, asthma, lung disease, spina bifida, injury, and chronic illness. In addition, results of meta-regression indicated that in comparison with the general population, WMDs of HUs in patients diagnosed with ear disease had significant differences.

On the basis of WMDs obtained by different valuation methods, we found that most pairs applied direct methods including VAS, SG, and TTO generated no significant difference between self- and proxy-reported HUs. And meta-regression showed that WMDs obtained by SG and TTO have no significant difference compared with those obtained by VAS, indicating the WMDs in HUs obtained using direct methods were similar. For indirect methods, both general instruments including HUI2, HUI3, and EQ-5D and children specific instruments including EQ-5D-Y and CHU-9D generated inconsistent WMDs in the direction and magnitude. Meta-regression indicated that the WMDs of HUs assessed by EQ-5D, HUI2, and PAHOM differed significantly from those assessed using the VAS instrument.

Regarding WMDs assessed by different proxy types, we found that parental proxy was dominantly used in the selected studies, and no obvious trend was found regarding the WMDs in HUs reported by this proxy. However, caregivers might underestimate the child’s HUs since all the related statistically significant WMD values were negative. On the contrary, clinicians might overestimate children’s HUs in cancer patients. Moreover, meta-regression demonstrated that WMDs obtained by the clinician and caregiver proxies were not significantly different from those obtained by parents.

## Discussion

This study was the first systematic and meta-analysis to compare self- and proxy-reported HUs quantitatively. The WMDs in HUs between self- and proxy-reports stratified by health conditions, valuation methods, and proxy types were estimated using meta-analysis, and the difference in WMDs between each health conditions, valuation methods, and proxy types were evaluated using meat-regressions. In general, disagreement exists between self- and proxy-reported HUs.

### Comparisons and implications

As regards proxy- versus self -reports specified by health conditions, consistent results were reported in some previous studies focused on specific health conditions. Jardine et al. [53] analyzed 21 studies including HRQoL data from both children with congenital health conditions and their parents, and concluded that a child’s perception of QoL differed from their parents, with parents frequently underestimating QoL. Pickard et al. [54] compared the HRQoL outcomes measured from children with acute lymphoblastic leukemia (ALL) and their parents, and they also found the responses of children and proxy were not interchangeable. Litsenburg et al. [55] included 15 studies to compare utility scores between self- and proxy-respondents for children with ALL, and the conclusion was that proxy-respondents were less reliable for observable conditions. Although these studies did not report specific differences, the fact that differences exist in self- and proxy-reported HUs between different health conditions were emphasized. Therefore, when researchers determine whether to include the self- or proxy-reported HUs, health conditions of the target populations should be one of important factors to consider.

For proxy- versus self-reports obtained by different valuation methods, Kwon et al*.* [16] performed a meta-regression and found that proxy-reported HUs were significantly higher when using HUI3 and VAS than self-reported HUs. In adult populations, significant differences in HUs were also found between direct and indirect valuation methods [56–58]. These evidences were consistent with our findings that valuation methods including direct and indirect instruments might cause a divergence between self- and proxy-reported HUs, direct valuation methods are straightforward and convenient for reseachers but not very comprehensible for respondents, indirect valuation methods are more complicated for reaseachers to calculate but easier for respondents to understand and answer. Researchers should take into full consideration about characteristics of different valuation methods for both direct and indirect ones. Hence, our study highlighted the importance of applying appropriate valuation methods, especially when it is necessary to measure the primary HUs, or to select the HUs obtained by different direct or indirect valuation methods for economic evaluations.

In terms of proxy- versus self-reports assessed by different proxy types, an earlier review conducted by Fluchel et al*.* [59] proved different proxy types can lead to differences in the inter-rater effects in HU estimates. Other studies showed that proxy-assessment by clinicians might lead to a discrepancy in HUs compared to proxy-assessment by parents [25, 32, 59]. It should be noted that meta-regression results showed no difference existing in WMDs assessed by caregivers or physicains versus parents, which does not mean that no difference presented in HUs assessed by proxy versus children themselves. Therefore, we suggest that the HUs to be assessed by children and proxies simultaneously, and researchers need to consider the HUs on the perspective from both self and proxy rather than just considering single perspective in order to obtain more comprehensive results. In addition, in order to avoid proxy-reports are from proxy’s personal judgment, clear instructions about incorporating the child’s perspective into assessment could be developed for all proxy types, which would improve the quality and accuracy of proxy-assessed HUs..

### Strengths and limitations

This study has two primary strengths. First, different from earlier reviews mainly used qualitative methods, our study included all the literatures which reported the HU values in pairwise self-proxy population to perform quantitative analyses, which can provide more direct evidence. WMD is used to estimate the difference in mean values between two groups, which is the suitable effect size for continuous data exactly as the HUs for this meta-analysis [20]. While meta-regression is commonly used to investigate heterogeneity for a meta-analysis, but it can also be used to investigate differences for categorical independent variables, so we applied meta-regression to assess the differences in WMDs among each potential factors [20]. Second, the differences in HUs were compared within and between each factor comprehensively. Since we not only compared the difference in HUs between self- and proxy-reports stratified by factors including health conditions, valuation methods, and proxy types, as well as to explore the divergence in WMDs between each factor by conducting meta-regression.

This study also has several limitations. First, when conducting meta-analysis, the heterogeneity was high (I^2^ > 50%) in certain results, which may be mainly caused by the target population coming from different studies. Although we tried our best to conduct sub-group analysis by taking health conditions, valuation methods, and proxy types into consideration, more specific sub-group analysis considering participant characteristics such as gender and age cannot be performed due to the lack of information. Second, when performing meta-regression, we adopted health conditions but not diagnoses as the independent variable, which made it unable to understand the differences in WMDs between each diagnoses, for the reason that limited sample size (211 pairs) cannot tolerate that many grouping variables. Third, the number of groups and associated sample sizes were relatively small for certain groups especially when specific to valuation methods under certain diagnoses, which may compromise the validity of our findings. Similarly, although meta-regression showed that differences in WMDs existed or not existed in certain group relative to reference group, the results may be biased given the small numbers of groups of interest. Fourth, funnel plots used to identify publication bias were not given in our study, for the reason that the funnel plots generated in our study cannot indicate publication bias, since certain pairs of self- and proxy-reports included in one single meta-analysis to estimate WMD were from same article. Fifth, the reasons why the differences existed among the valuation measures or the proxy types cannot be addressed by conducting this meta-analyses. Therefore, we cannot suggest which valuation method or proxy type is most reliable based on our current results. Lastly, our systematic review only included studies published in the English language.

## Conclusions

Assessment of children’s HUs which can be measured by both self and proxy is important not only for HRQoL researches but also for economic evaluations such as cost-utility/effectiveness analysis regarding children population. However, the way to measure and cite children’s HUs in relative studies has been a challenge for current researchers due to the lack of quantitative analysis comparing differences between proxy- and self-reported HUs. The results of our study show that differences indeed exist in HUs between self- and proxy-reports, and potential factors including health conditions, valuation methods, and proxy types impact the direction and magnitude of the differences in different ways. These findings may provide certain guidance for measuring primay children’s HUs, or be an available source for conducting economic evaluations regarding children population. In addition, due to the paucity and heterogeneity of existing studies, there are inevitable bias in our study, so future studies are needed to synthesize more evidence and to explore the trend of differences.

## Supplementary Information

**Additional file 1.** Appendix 1: PRISMA checklist. Appendix 2: Search terms and strategy. Appendix 3: Risk of bias assessment by Newcastle Ottawa Scale. Appendix 4: Weighted mean differences in health utilities between self- and proxy-reports by health conditions and valuation methods. Appendix 5: Weighted mean differences in health utilities between self- and proxy-reports by health conditions and proxy types.

## Data Availability

Not applicable.

## Abbreviations

Hus: Health utilities
WMDs: Weighted mean differences
CEA: Cost-effectiveness analysis
CBA: Cost–benefit analysis
CUA: Cost-utility analysis
QALYs: Quality-adjusted life years
PRISMA: Preferred reporting items for systematic reviews and meta-analyses
ICD-10: International classification of diseases 10 revision
MAUIs: Multi-attribute utility instruments
VAS: Visual analogue scale
SG: Standard gamble
TTO: Time trade-off
CHU9D: Child health utility 9-dimension
HUI: Health utility index
PAHOM: Pediatric asthma health outcome measure
ALL: Acute lymphoblastic leukemia
VLBW/VP: Very low birth weight/very preterm
ELBW/EP: Extremely low birth weight/extremely preterm
ODD: Oppositional defiant disorder
CD: Conduct disorder
DBD: Disruptive behaviour disorder
ADHD: Attention deficit hyperactivity disorder
SA: Substance abuse
SD: Standard deviation
CI: Confidence intervals
