# Supplementary material for: A comparison of self-reported and proxy-reported health utilities in children: a systematic review and meta-analysis
Source: Health Qual Life Outcomes. 2021 Feb 5;19:45. doi: 10.1186/s12955-021-01677-0 (PMC7866432; doi:10.1186/s12955-021-01677-0)
Supplement: Supplementary file 1 — Additional file 1. Appendix 1: PRISMA checklist. Appendix 2: Search terms and strategy. Appendix 3: Risk of bias assessment by Newcastle Ottawa Scale. Appendix 4: Weighted mean differences in health utilities between self- and proxy-reports by health conditions and valuation methods. Appendix 5: Weighted mean differences in health utilities between self- and proxy-reports by health conditions and proxy types. [file 12955_2021_1677_MOESM1_ESM.docx]

**Appendix list**

# Appendix 1: PRISMA checklist

| **Section/topic** | **#** | **Checklist item** | **Reported on page #** |
| --- | --- | --- | --- |
| **TITLE** | | |  |
| Title | 1 | Identify the report as a systematic review, meta-analysis, or both. | P1 |
| **ABSTRACT** | | |  |
| Structured summary | 2 | Provide a structured summary including, as applicable: background; objectives; data sources; study eligibility criteria, participants, and interventions; study appraisal and synthesis methods; results; limitations; conclusions and implications of key findings; systematic review registration number. | P2 |
| **INTRODUCTION** | | |  |
| Rationale | 3 | Describe the rationale for the review in the context of what is already known. | P3 |
| Objectives | 4 | Provide an explicit statement of questions being addressed with reference to participants, interventions, comparisons, outcomes, and study design (PICOS). | P4 |
| **METHODS** | | |  |
| Protocol and registration | 5 | Indicate if a review protocol exists, where it can be accessed, and, if available, provide registration information including registration number. | NA |
| Eligibility criteria | 6 | Specify study characteristics (e.g., PICOS, length of follow-up) and report characteristics (e.g., years considered, language, publication status) used as criteria for eligibility, giving rationale. | P4 |
| Information sources | 7 | Describe all information sources (e.g., databases with dates of coverage, contact with study authors to identify additional studies) in the search and date last searched. | P4 |
| Search | 8 | Present full electronic search strategy for at least one database, including any limits used, such that it could be repeated. | Appendix |
| Study selection | 9 | State the process for selecting studies (i.e., screening, eligibility, included in systematic review, and, if applicable, included in the meta-analysis). | P4 |
| Data collection process | 10 | Describe method of data extraction (e.g., piloted forms, independently, in duplicate) and any processes for obtaining and confirming data from investigators. | P4 |
| Data items | 11 | List and define all variables for which data were sought (e.g., PICOS, funding sources) and any assumptions and simplifications made. | P4 |
| Risk of bias in individual studies | 12 | Describe methods used for assessing risk of bias of individual studies (including specification of whether this was done at the study or outcome level), and how this information is to be used in any data synthesis. | P4~P5 |
| Summary measures | 13 | State the principal summary measures (e.g., risk ratio, difference in means). | P5 |
| Synthesis of results | 14 | Describe the methods of handling data and combining results of studies, if done, including measures of consistency (e.g., I^2^) for each meta-analysis. | P5 |
| Risk of bias across studies | 15 | Specify any assessment of risk of bias that may affect the cumulative evidence (e.g., publication bias, selective reporting within studies). | P5 |
| Additional analyses | 16 | Describe methods of additional analyses (e.g., sensitivity or subgroup analyses, meta-regression), if done, indicating which were pre-specified. | P5 |
| **RESULTS** | | |  |
| Study selection | 17 | Give no. studies screened, assessed for eligibility, and included in the review, with reasons for exclusions at each stage, ideally with a flow diagram. | P5, Figure 1 |
| Study characteristics | 18 | For each study, present characteristics for which data were extracted (e.g., study size, PICOS, follow-up period) and provide the citations. | P5~P6, Table 1 |
| Risk of bias within studies | 19 | Present data on risk of bias of each study and, if available, any outcome level assessment (see item 12). | P6, Appendix |
| Results of individual studies | 20 | For all outcomes considered (benefits or harms), present, for each study: (a) simple summary data for each intervention group (b) effect estimates and confidence intervals, ideally with a forest plot. | P6~P7, Table 2, Table 3,  Appendix |
| Synthesis of results | 21 | Present results of each meta-analysis done, including confidence intervals and measures of consistency. |  |
| Risk of bias across studies | 22 | Present results of any assessment of risk of bias across studies (see Item 15). |  |
| Additional analysis | 23 | Give results of additional analyses, if done (e.g., sensitivity or subgroup analyses, meta-regression [see Item 16]). |  |
| **DISCUSSION** | | |  |
| Summary of evidence | 24 | Summarize the main findings including the strength of evidence for each main outcome; consider their relevance to key groups | P8~P9 |
| Limitations | 25 | Discuss limitations at study and outcome level (e.g., risk of bias), and at review-level (e.g., incomplete retrieval of identified research, reporting bias). | P9 |
| Conclusions | 26 | Provide a general interpretation of the results in the context of other evidence, and implications for future research. | P8~P9 |
| **FUNDING** | | |  |
| Funding | 27 | Describe sources of funding for the systematic review and other support (e.g., supply of data); role of funders for the systematic review. | NA |

# Appendix 2: Search terms and strategy

**Health-related quality of life**

1 "Quality of life" or "preference-based quality of life"

2 Utility or utilities or cost utility or cost-utility or cost-effectiveness or cost effectiveness

3 EQ-5D or "EQ 5D" or EQ5D or EQ-5D-Y or "EQ 5D Y"

4 Short-form survey-6D or short form 6D or SF-6D or "SF 6D" or SF6D

5 "Health utilities index"

6 "Quality of well being" or "quality of well-being" or QWB

7 16D Health-Related Quality of Life or 16D HRQoL or 17D Health-Related Quality of Life or 17D HRQoL

8 AQoL-6D or Assessment of Quality of Life-6D

9 "Child Health Utility 9 Dimension" or CHU9D or CHU-9D or "CHU 9D"

10 Adolescent Health Utility Measure or AHUM

11 15-dimensional instrument or 15 dimensional instrument

12 Preference-based measure of HRQoL or preference based measure of HRQoL

13 Multi-attribute utility instrument or multiattribute utility instrument

14 Standard Gamble or standard-gamble

15 Time trade off or time trade-off

16 Best worst scaling or best-worst scaling

17 Discrete choice experiment or discrete-choice experiment

18 Person trade off or person trade-off

**Pediatric**

19 Pediatri* or paediatri* or child or children or childhood or adolesc* or kid or kids or teen* or youth* or infant* or newborn* or neonat*

**Proxy**

20 Proxy or proxies or parent* or physician* or nurse* or teacher* or caregiver*

21 OR: 1~18

**Final search: 19 AND 20 AND 21**

| **Search** | **PubMed** | **Embase** | **Web of Science** | **Cochrane Library** |
| --- | --- | --- | --- | --- |
| **Utility Terms** | ((((((((((((((((((("Quality of life"[Title/Abstract] OR "preference-based quality of life"[Title/Abstract])) OR (Utility[Title/Abstract] OR utilities[Title/Abstract] OR cost utility[Title/Abstract] OR cost-utility[Title/Abstract] OR cost-effectiveness[Title/Abstract] OR cost effectiveness[Title/Abstract])) OR (EQ-5D[Title/Abstract] OR "EQ 5D"[Title/Abstract] OR EQ5D[Title/Abstract] OR EQ-5D-Y[Title/Abstract] OR "EQ 5D Y"[Title/Abstract])) OR (Short-form survey-6D[Title/Abstract] OR short form 6D[Title/Abstract] OR SF-6D[Title/Abstract] OR "SF 6D"[Title/Abstract] OR SF6D[Title/Abstract])) OR "Health utilities index"[Title/Abstract]) OR ("Quality of well being"[Title/Abstract] OR "quality of well-being"[Title/Abstract] OR QWB[Title/Abstract])) OR (16D Health-Related Quality of Life[Title/Abstract] OR 16D HRQoL[Title/Abstract] OR 17D Health-Related Quality of Life[Title/Abstract] OR 17D HRQoL[Title/Abstract])) OR (AQoL-6D[Title/Abstract] OR Assessment of Quality of Life-6D[Title/Abstract])) OR ("Child Health Utility 9 Dimension"[Title/Abstract] OR CHU9D[Title/Abstract] OR CHU-9D[Title/Abstract] OR "CHU 9D"[Title/Abstract])) OR (Adolescent Health Utility Measure[Title/Abstract] OR AHUM[Title/Abstract])) OR (15-dimensional instrument[Title/Abstract] OR 15 dimensional instrument[Title/Abstract])) OR (Preference-based measure of HRQoL[Title/Abstract] OR preference based measure of HRQoL[Title/Abstract])) OR (Multi-attribute utility instrument[Title/Abstract] OR multiattribute utility instrument[Title/Abstract])) OR (Standard Gamble[Title/Abstract] OR standard-gamble[Title/Abstract])) OR (Time trade off[Title/Abstract] OR time trade-off[Title/Abstract])) OR (Best worst scaling[Title/Abstract] OR best-worst scaling[Title/Abstract])) OR (Discrete choice experiment[Title/Abstract] OR discrete-choice experiment[Title/Abstract])) OR (Person trade off[Title/Abstract] OR person trade-off[Title/Abstract])) | '('quality of life':ab,ti OR 'preference-based quality of life':ab,ti OR utility:ab,ti OR utilities OR 'cost utility':ab,ti OR 'cost effectiveness':ab,ti OR 'eq 5d':ab,ti OR 'eq5d':ab,ti OR eq5d:ab,ti OR 'eq 5d y':ab,ti OR 'short-form survey-6d':ab,ti OR 'short form 6d':ab,ti OR 'sf 6d':ab,ti OR sf6d:ab,ti OR 'health utilities index':ab,ti OR 'quality of well being':ab,ti OR 'quality of well-being':ab,ti OR qwb:ab,ti OR '16d health-related quality of life':ab,ti OR '16d hrqol':ab,ti OR '17d health-related quality of life':ab,ti OR '17d hrqol':ab,ti OR 'aqol 6d':ab,ti OR 'assessment of quality of life-6d':ab,ti OR 'child health utility 9 dimension':ab,ti OR chu9d:ab,ti OR 'chu 9d':ab,ti OR 'adolescent health utility measure':ab,ti OR ahum:ab,ti OR '15-dimensional instrument':ab,ti OR '15 dimensional instrument':ab,ti OR 'preference-based measure of hrqol':ab,ti OR 'preference based measure of hrqol':ab,ti OR 'multi-attribute utility instrument':ab,ti OR 'multiattribute utility instrument':ab,ti OR 'standard gamble':ab,ti OR 'time trade off':ab,ti OR 'time trade-off':ab,ti OR 'best worst scaling':ab,ti OR 'best-worst scaling':ab,ti OR 'discrete choice experiment':ab,ti OR 'discrete-choice experiment':ab,ti OR 'person trade off':ab,ti OR 'person trade-off':ab,ti) | TS=( "Quality of life" OR "preference-based quality of life" OR Utility OR utilities OR cost utility OR cost-utility OR cost-effectiveness OR cost effectiveness OR EQ-5D OR "EQ 5D" OR EQ5D OR EQ-5D-Y OR "EQ 5D Y" OR Short-form survey-6D OR short form 6D OR SF-6D OR "SF 6D" OR SF6D OR "Health utilities index" OR "Quality of well being" OR "quality of well-being" OR QWB 16D Health-Related Quality of Life OR 16D HRQoL OR 17D Health-Related Quality of Life OR 17D HRQoL OR AQoL-6D OR Assessment of Quality of Life-6D OR "Child Health Utility 9 Dimension" OR CHU9D OR CHU-9D OR "CHU 9D" OR Adolescent Health Utility Measure OR AHUM OR 15-dimensional instrument OR 15 dimensional instrument OR Preference-based measure of HRQoL OR preference based measure of HRQoL OR Multi-attribute utility instrument OR multiattribute utility instrument OR Standard Gamble OR standard-gamble OR Time trade off OR time trade-off OR Best worst scaling OR best-worst scaling OR Discrete choice experiment OR discrete-choice experiment OR Person trade off OR person trade-off) | (Quality of life OR preference-based quality of life OR Utility OR utilities OR cost utility OR cost-utility OR cost-effectiveness OR cost effectiveness OR EQ-5D OR EQ 5D OR EQ5D OR EQ-5D-Y OR EQ 5D Y OR Short-form survey-6D OR short form 6D OR SF-6D OR SF 6D OR SF6D OR Health utilities index OR Quality of well being OR quality of well-being OR QWB OR 16D Health-Related Quality of Life OR 16D HRQoL OR 17D Health-Related Quality of Life OR 17D HRQoL OR AQoL-6D OR Assessment of Quality of Life-6D OR Child Health Utility 9 Dimension OR CHU9D OR CHU-9D OR CHU 9D OR Adolescent Health Utility Measure OR AHUM OR 15 dimensional instrument OR 15 dimensional instrument OR Preference-based measure of HRQoL OR preference based measure of HRQoL OR Multi-attribute utility instrument OR multiattribute utility instrument OR Standard Gamble OR standard-gamble OR Time trade off OR time trade-off OR Best worst scaling OR best-worst scaling OR Discrete choice experiment OR discrete-choice experiment OR Person trade off OR person trade-off in Title Abstract Keyword) |
| **Childhood Terms** | (Pediatri*[Title/Abstract] OR paediatri*[Title/Abstract] OR child[Title/Abstract] OR children[Title/Abstract] OR childhood[Title/Abstract] OR adolesc*[Title/Abstract] OR kid[Title/Abstract] OR kids[Title/Abstract] OR teen*[Title/Abstract] OR youth*[Title/Abstract] OR infant*[Title/Abstract] OR newborn*[Title/Abstract] OR neonat*[Title/Abstract]) | (pediatri*:ab,ti OR paediatri*:ab,ti OR 'child':ab,ti OR child:ab,ti OR 'children':ab,ti OR children:ab,ti OR 'childhood':ab,ti OR childhood:ab,ti OR adolesc*:ab,ti OR kid:ab,ti OR kids:ab,ti OR teen*:ab,ti OR youth*:ab,ti OR infant*:ab,ti OR newborn*:ab,ti OR neonat*:ab,ti) | TS=( Pediatri* OR paediatri* OR child OR children OR childhood OR adolesc* OR kid OR kids OR teen* OR youth* OR infant* OR newbORn* OR neonat*) | (Pediatri* OR paediatri* OR child OR children OR childhood OR adolesc* OR kid OR kids OR teen* OR youth* OR infant* OR newborn* OR neonat* in Title Abstract Keyword) |
| **Proxy Terms** | (Proxy[Title/Abstract] OR proxies[Title/Abstract] OR parent*[Title/Abstract] OR physician*[Title/Abstract] OR nurse*[Title/Abstract] OR teacher*[Title/Abstract] OR caregiver*[Title/Abstract]) | ('proxy':ab,ti OR proxies:ab,ti OR parent*:ab,ti OR physician*:ab,ti OR nurse*:ab,ti OR teacher*:ab,ti OR caregiver*:ab,ti) | TS=( Proxy OR proxies OR parent* OR physician* OR nurse* OR teacher* OR caregiver*) | (Proxy or proxies or parent* OR physician* OR nurse* OR teacher* OR caregiver* in Title Abstract Keyword) |
| **Results** | 13,939 | 22,698 | 31,922 | 2,880 |

# Appendix 3: Risk of bias assessment by Newcastle Ottawa Scale

| **Author** (**Year**) | **Study Design** | **Selection** (**Max 5**) | | | | **Comparability** (**Max 2**) | **Outcome** (**Max 3**) | | | |  |
| --- | --- | --- | --- | --- | --- | --- | --- | --- | --- | --- | --- |
|  |  | **Representativeness of the sample** | **Sample size** | **Non**-**respondents** | **Ascertainment of the exposure** |  | **Assessment of the outcome** | | **Statistical test** | | **Total score**  **(Max: 10)** |
| Verrips(2001) | C | * | * | * | * | ** | * | | * | | 8 |
| Wolke(2013) | C | * | * |  | * | ** | * | | * | | 7 |
| Saigal(1999) | C | * | * | * | * | ** | * | | * | | 8 |
| Lee(2011) | C |  | * | * | * | ** | * | | * | | 7 |
| Kulpeng(2013) | C | * |  | * | * | ** | * | |  | | 6 |
| Trent(2011) | C | * | * | * | * | ** | * | | * | | 8 |
| Jelsma(2010) | C |  | * |  | ** | ** | * | | * | | 7 |
| Vermeulen(2017) | C | * |  | * | * | ** | * | |  | | 6 |
| Hanberger(2009) | C | * | * | * | * | ** | * | | * | | 8 |
| Rhodes(2012) | C | * | * | * | * | ** | * | | * | | 8 |
| Sims(2017) | C | * | * | * | * | ** | * | | * | | 8 |
| Medeiros(2019) | C | * | * | * | * | ** | * | | * | | 8 |
| Kirkham(2019) | C | * | * | * | ** | ** | * | | * | | 9 |
| Lopez (2019) | C | * | * |  | ** | ** | * | | * | | 8 |
| Bray(2017） | C | * |  | * | ** | ** | * | | * | | 8 |
| **Author** (**Year**) | **Study Design** | **Selection** (**Max 4**) | | | | **Comparability** (**Max 2**) | **Outcome** (**Max 3**) | | | |  |
|  |  | **Representativeness of the exposed cohort** | **Selection of the non-exposed cohort** | **Ascertainment of exposure** | **Demonstration that outcome of interest was not present at start** |  | **Assessment of outcome** | **Was follow**-**up long enough for outcomes to occur** | | **Adequacy of follow up of cohorts** | **Total score**  **(Max: 9)** |
| Penn(2011) | L | * | * | * | * | ** | * | * | | * | 9 |
| Brunner(2004) | L | * | * | * | * | ** | * |  | | * | 8 |
| Baumann(2016) | L | * | * | * | * | ** | * | * | | * | 9 |
| Robertson(2016) | L | * | * | * | * | ** | * | * | | * | 9 |
| Perez-Sousa(2018） | L | * | * | * | * | ** | * | * | | * | 9 |
| Creswell(2017) | L | * | * | * | * | ** | * | * | | * | 9 |
| Shi(2017) | L | * | * | * | * | ** | * |  | | * | 8 |
| **Author** (**Year**) | **Study Design** | **Selection** (**Max 4**) | | | | **Comparability** (**Max 2**) | **Exposure** (**Max 3**) | | | |  |
|  |  | **Is the case definition adequate?** | **Representativeness of the cases** | **Selection of Controls** | **Definition of Controls** |  | **Ascertainment of exposure** | **Same method of ascertainment for cases and controls** | | **Non-Response rate** | **Total score**  **(Max: 9)** |
| Cardarelli(2006) | CS |  | * | * | * | ** | * | * | |  | 7 |
| Fu(2006) | CS | * | * |  | * | ** | * | * | |  | 7 |
| Glaser(1999) | CS | * | * |  | * | ** | * | * | |  | 7 |
| Belfort(2011) | CS | * | * | * | * | ** | * | * | |  | 8 |
| Brunner(2003) | CS | * | * | * | * | ** | * | * | | * | 9 |
| Czyzewski(1994) | CS | * | * | * | * | ** | * | * | | * | 9 |
| Gerald(2012) | CS | * | * |  | * | ** | * | * | |  | 7 |
| Sung(2004) | CS |  | * | * | * | ** | * | * | | * | 8 |
| C: Cross-sectional; L: Longitudinal; CS: Case series | | | | | | | | | | |  |

# Appendix 4: Weighted mean differences in health utilities between self- and proxy-reports by health conditions and valuation methods

| **Health conditions*** | **Valuation method** | **N groups (N participants)** | **N groups (N proxies)** | **WMD† (95% CI)** | ***P*** | **I^2^** |
| --- | --- | --- | --- | --- | --- | --- |
| **General populations** | VAS | 3(1875) | 3(1875) | 0.041(0.019, 0.064) | <0.001 | 83.1% |
|  | HUI3 | 6(871) | 6(871) | 0.029 (0.016, 0.042) | <0.001 | 60.2% |
| **Infectious and parasitic diseases** |  |  |  |  |  |  |
| Bacteremia | VAS | 1(9) | 1(7) | 0.030(-0.044, 0.104) | 0.428 | / |
|  | EQ-5D | 1(9) | 1(7) | 0.040(-0.096, 0.176) | 0.566 | / |
|  | HUI2 | 1(9) | 1(7) | -0.080(-0.172, 0.012) | 0.090 | / |
|  | HUI3 | 1(9) | 1(7) | 0.070(-0.062, 0.202) | 0.300 | / |
| Meningitis | VAS | 1(7) | 1(12) | -0.040(-0.124, 0.044) | 0.352 | / |
|  | EQ-5D | 1(7) | 1(12) | -0.230(-0.383, -0.077) | 0.003 | / |
|  | HUI2 | 1(7) | 1(12) | -0.140(-0.216, -0.064) | <0.001 | / |
|  | HUI3 | 1(7) | 1(12) | -0.160(-0.304, -0.016) | 0.030 | / |
| **Cancer** |  |  |  |  |  |  |
| Brain tumour | HUI2 | 4(95) | 4(125) | 0.032(-0.044, 0.108) | 0.411 | 58.8% |
|  | HUI3 | 4(99) | 4(107) | 0.085(-0.036, 0.206) | 0.171 | 69.7% |
| Leukaemia/Lymphoma | HUI2 | 1(33) | 1(79) | -0.040(--0.108, 0.028) | 0.251 | 0.0% |
| Medulloblastoma | HUI2 | 2(76) | 2(76) | -0.004(-0.058, 0.050) | 0.892 | 0.0% |
|  | HUI3 | 2(76) | 2(76) | -0.039(-0.131, 0.053) | 0.405 | 0.0% |
| Mixed cancer diagnosis | HUI2 | 4(261) | 4(590) | -0.010(-0.041, 0.020) | 0.509 | 60.6% |
|  | HUI3 | 2(350) | 2(401) | 0.186(0.137, 0.235) | <0.001 | 57.0% |
| **Endocrine, nutritional and metabolic disorders** |  |  |  |  |  |  |
| Diabetes | VAS | 5(801) | 5(801) | -0.005(-0.018, 0.009) | 0.499 | 0.0% |
|  | TTO | 1(95) | 1(221) | 0.030(-0.030, 0.090) | 0.331 | / |
|  | EQ-5D | 1(148) | 1(151) | 0.000(-0.023, 0.023) | 1.000 | / |
|  | HUI3 | 2(338) | 2(330) | 0.020(0.003, 0.037) | 0.021 | 0.0% |
| Overweight or obese | VAS | 10(615) | 10(615) | 0.058(0.037, 0.079) | <0.001 | 94.9% |
|  | EQ-5D-Y | 6(313) | 6(313) | 0.046(0.033, 0.060) | <0.001 | 85.6% |
|  | HUI3 | 2(152) | 2(152) | 0.093(0.084, 0.103) | <0.001 | 31.2% |
| Cystic fibrosis | QWB | 1(55) | 1(199) | 0.030(0.005, 0.055) | 0.017 | / |
| **Mental and behavioral disorders‡** |  |  |  |  |  |  |
| Anxiety disorders | CHU-9D | 6(339) | 6(310) | 0.011(-0.001, 0.024) | 0.077 | 0.0% |
| CD | VAS | 3(86) | 3(46) | 0.085(-0.113, 0.282) | 0.400 | 94.2% |
| CD+SA | VAS | 3(86) | 3(46) | 0.085(-0.043, 0.213) | 0.193 | 84.1% |
| CD+ADHD | VAS | 3(86) | 3(46) | 0.075(-0.096, 0.247) | 0.390 | 91.8% |
| DBD | VAS | 3(86) | 3(46) | 0.072(-0.110, 0.254) | 0.438 | 93.3% |
| DBD+SA | VAS | 3(86) | 3(46) | 0.055(-0.068, 0.177) | 0.382 | 84.2% |
| DBD+ADHD | VAS | 3(86) | 3(46) | 0.052(-0.072, 0.176) | 0.412 | 83.9% |
| ODD | VAS | 3(86) | 3(46) | 0.101(-0.045, 0.246) | 0.174 | 90.5% |
| ODD+SA | VAS | 3(86) | 3(46) | 0.121(-0.050, 0.291) | 0.167 | 89% |
| ODD+ADHD | VAS | 3(86) | 3(46) | 0.091(-0.127, 0.310) | 0.414 | 95% |
| Paediatric mobility impairment | VAS | 3(26) | 3(26) | -0.036(-0.116, 0.045) | 0.382 | 0.0% |
|  | EQ-5D-Y | 3(22) | 3(22) | -0.234(-0.438, -0.030) | 0.024 | 63.3% |
|  | HUI2 | 3(26) | 3(26) | -0.043(-0.087, 0.000) | 0.050 | 0.0% |
|  | HUI3 | 3(26) | 3(26) | -0.060(-0.112, -0.008) | 0.023 | 0.0% |
| Prolonged acute convulsive seizures | EQ-5D | 2(54) | 2(556) | -0.265(-0.344, -0.186) | <0.001 | 0.0% |
| **Nervous system disorders** |  |  |  |  |  |  |
| Epilepsy | VAS | 1(16) | 1(4) | -0.010(-0.066, 0.046) | 0.725 | / |
|  | EQ-5D | 1(16) | 1(4) | 0.000(-0.065, 0.065) | 1.000 | / |
|  | HUI2 | 1(16) | 1(4) | -0.010(-0.066, 0.046) | 0.725 | / |
|  | HUI3 | 1(16) | 1(4) | -0.020(-0.100, 0.060) | 0.624 | / |
| **Diseases of the ear** |  |  |  |  |  |  |
| Hearing loss | VAS | 1(15) | 1(7) | -0.030(-0.068, 0.008) | 0.120 | / |
|  | EQ-5D | 1(15) | 1(7) | 0.070(0.032, 0.108) | <0.001 | / |
|  | HUI2 | 1(15) | 1(7) | 0.080(0.033, 0.127) | 0.001 | / |
|  | HUI3 | 1(15) | 1(7) | 0.170(0.096, 0.244) | <0.001 | / |
| Otitis media | VAS | 1(7) | 1(11) | -0.050(-0.118, 0.018) | 0.152 | / |
|  | EQ-5D | 1(7) | 1(11) | 0.000(-0.065, 0.065) | 1.000 | / |
|  | HUI2 | 1(7) | 1(11) | 0.120(0.052, 0.188) | 0.001 | / |
|  | HUI3 | 1(7) | 1(11) | 0.200(0.097, 0.303) | <0.001 | / |
| **Circulatory system disorders** |  |  |  |  |  |  |
| Stroke | VAS | 1(22) | 1(22) | -0.020(-0.115, 0.075) | 0.678 | / |
|  | SG | 1(22) | 1(22) | 0.040(-0.129, 0.209) | 0.642 | / |
| **Respiratory system disorders** |  |  |  |  |  |  |
| Asthma | VAS | 1(22) | 1(22) | -0.020(-0.136, 0.096) | 0.734 | / |
|  | SG | 1(22) | 1(22) | 0.040(-0.026, 0.106) | 0.235 | / |
|  | PAHOM | 30(2806) | 30(2806) | -0.035(-0.040, -0.030) | <0.001 | 31.6% |
| Lung disease | VAS | 2(20) | 2(20) | -0.043(-0.087, 0.001) | 0.054 | 0.0% |
|  | EQ-5D | 2(20) | 2(20) | -0.052(-0.178, 0.075) | 0.424 | 68.7% |
|  | HUI2 | 2(20) | 2(20) | -0.016(-0.066, 0.033) | 0.517 | 0.0% |
|  | HUI3 | 2(20) | 2(20) | 0.014(-0.056, 0.084) | 0.691 | 0.0% |
| **Musculoskeletal disorders** |  |  |  |  |  |  |
| MSKD | VAS | 8(371) | 8(517) | 0.030(-0.005, 0.066) | 0.095 | 50.4% |
|  | SG | 7(196) | 7(315) | 0.015(-0.010, 0.040) | 0.249 | 45.5% |
|  | HUI3 | 1(45) | 1(68) | -0.034(-0.128, 0.060) | 0.480 | / |
| **Genitourinary system disorders** |  |  |  |  |  |  |
| Pelvic inflammatory disease | VAS | 5(670) | 5(605) | 0.146(0.119, 0.714) | <0.001 | 5.9% |
|  | TTO | 5(670) | 5(605) | 0.085(0.051, 0.120) | <0.001 | 0.0% |
| **Conditions originating in the perinatal period** |  |  |  |  |  |  |
| ELBW/EP | SG | 4(1056) | 4(1100) | 0.065(0.023, 0.106) | 0.002 | 45.8% |
| VLBW/VP | HUI3 | 6(1053) | 6(1053) | 0.009(-0.016, 0.034) | 0.481 | 98.9% |
| **Congenital malformations** |  |  |  |  |  |  |
| Spina bifida | VAS | 1(62) | 1(66) | -0.061(-0.072, -0.050) | <0.001 | / |
|  | HUI3 | 1(63) | 1(66) | -0.026(-0.040, -0.012) | <0.001 | / |
| **Injury, poisoning and other consequences of external causes** |  |  |  |  |  |  |
| Injury | VAS | 1(22) | 1(22) | -0.090(-0.167, -0.013) | 0.022 | / |
|  | SG | 1(22) | 1(22) | -0.040(-0.239, 0.159) | 0.694 | / |
| **Chronic illness** |  |  |  |  |  |  |
| Chronic illness | VAS | 2(82) | 2(82) | -0.035(-0.089, 0.018) | 0.196 | 0.0% |
|  | SG | 1(22) | 1(22) | 0.000(-0.056, 0.056) | 1.000 | / |
|  | TTO | 1(22) | 1(22) | -0.150(-0.287, -0.013) | 0.032 | / |
|  | HUI2 | 1(19) | 1(19) | -0.130(-0.234, -0.026) | 0.014 | / |
|  | HUI3 | 1(19) | 1(19) | -0.130(-0.254, -0.006) | 0.039 | ./ |
| ODD: Oppositional defiant disorder; CD: Conduct disorder; DBD: Disruptive behaviour disorder; ADHD: Attention deficit hyperactivity disorder; SA: Substance abuse; VLBW/VP: Very low birth weight/ Very preterm; ELBW/EP: Extremely low birth weight/ Extremely preterm.  VAS: Visual analogue scale; SG: Standard gamble; TTO: Time trade-off; HUI: Health utility index; EQ-5D-Y: EuroQol 5-dimension youth version; CHU9D: Child health utility 9-dimension; QWB: Quality of well-being scale; PAHOM: Pediatric asthma health outcome measure.  *Health conditions were reported based on ICD-10 categories.  †Proxy-reported health utilities minus self-reported health utilities.  ‡Plus indicates patients have multiple diagnoses. | | | | | | |

# Appendix 5: Weighted mean differences in health utilities between self- and proxy-reports by health conditions and proxy types

| **Health conditions*** | **Proxy type** | **N groups**  **(N participants)** | **N groups**  **(N proxies)** | **WMD† (95% CI)** | ***P*** | **I^2^** |
| --- | --- | --- | --- | --- | --- | --- |
| **General population** | parents | 9(2736) | 9(2746) | 0.034(0.021, 0.047) | <0.001 | 75.5% |
| **Infectious and parasitic diseases** |  |  |  |  |  |  |
| Bacteremia | caregivers | 4(36) | 4(28) | 0.005(-0.044, 0.055) | 0.829 | 38.4% |
| Meningitis | caregivers | 4(28) | 4(48) | -0.117(-0.167, -0.067) | <0.001 | 49.9% |
| **Cancer** |  |  |  |  |  |  |
| Brain tumour | parents | 4(92) | 4(129) | -0.007 (-0.114, 0.101) | 0.900 | 67.1% |
|  | clinicians | 4(102) | 4(103) | 0.104(0.054, 0.155) | <0.001 | 0.0% |
| Leukaemia/Lymphoma | parents | 1(33) | 1(79) | -0.040(-0.108, 0.028) | 0.251 | / |
| Medulloblastoma | parents | 4(152) | 4(152) | -0.013(-0.059, 0.034) | 0.589 | 0.0% |
| Mixed cancer diagnosis | parents | 4(461) | 4(590) | -0.010(-0.041, 0.020) | 0.509 | 60.6% |
|  | clinicians | 2(350) | 2(401) | 0.186(0.137, 0.235) | <0.001 | 57.0% |
| **Endocrine, nutritional and metabolic disorders** |  |  |  |  |  |  |
| Diabetes | parents | 4(581) | 4(702) | 0.014(0.000, 0.027) | 0.045 | 0.0% |
|  | caregivers | 5(801) | 5(801) | -0.005(-0.018, 0.009) | 0.499 | 0.0% |
| Overweight or obese | parents | 18(1080) | 18(1080) | 0.059(0.045, 0.073) | <0.001 | 94.9% |
| Cystic fibrosis | parents | 1(55) | 1(199) | 0.030(0.005, 0.055) | 0.017 | / |
| **Mental and behavioral disorders** |  |  |  |  |  |  |
| Anxiety disorders | parents | 6(339) | 6(310) | 0.011 (-0.001, 0.024) | 0.077 | 0.0% |
| CD | parents | 3(86) | 3(46) | 0.085(-0.113, 0.282) | 0.400 | 94.2% |
| CD+SA | parents | 3(86) | 3(46) | 0.085 (-0.043, 0.213) | 0.193 | 84.1% |
| CD+ADHD | parents | 3(86) | 3(46) | 0.075(-0.096, 0.247) | 0.390 | 91.8% |
| DBD | parents | 3(86) | 3(46) | 0.072(-0.110, 0.254) | 0.438 | 93.3% |
| DBD+SA | parents | 3(86) | 3(46) | 0.055(-0.068, 0.177) | 0.382 | 84.2% |
| DBD+ADHD | parents | 3(86) | 3(46) | 0.052(-0.072, 0.176) | 0.412 | 83.9% |
| ODD | parents | 3(86) | 3(46) | 0.101(-0.045, 0.246) | 0.174 | 90.5% |
| ODD+SA | parents | 3(86) | 3(46) | 0.121(-0.050, 0.291) | 0.167 | 89.0% |
| ODD+ADHD | parents | 3(86) | 3(46) | 0.091(-0.127, 0.310) | 0.414 | 95.0% |
| Paediatric mobility impairment | parents | 12(100) | 12(100) | -0.058(-0.087, -0.028) | <0.001 | 7.6% |
| Prolonged acute convulsive seizures | parents | 1(27) | 1(279) | -0.270 (-0.382, -0.158) | <0.001 | / |
|  | clinicians | 1(27) | 1(277) | -0.260(-0.373, -0.147) | <0.001 | / |
| **Nervous system disorders** |  |  |  |  |  |  |
| Epilepsy | caregivers | 4(64) | 4(16) | 0.009(-0.040, 0.022) | 0.560 | 0.0% |
| **Diseases of the ear** |  |  |  |  |  |  |
| Hearing loss | caregivers | 4(60) | 4(28) | 0.068(-0.005, 0.141) | 0.067 | 90.0% |
| Otitis media | caregivers | 4(28) | 4(44) | 0.063(-0.039, 0.165) | 0.225 | 86.8% |
| **Circulatory system disorders** |  |  |  |  |  |  |
| Stroke | parents | 2(44) | 2(44) | -0.006(-0.088, 0.077) | 0.893 | 0.0% |
| **Respiratory system disorders** |  |  |  |  |  |  |
| Asthma | parents | 32(2850) | 32(2850) | -0.034(-0.039, -0.029) | <0.001 | 34.6% |
| Lung disease | caregivers | 8(80) | 8(80) | -0.030(-0.057, -0.003) | 0.030 | 0.7% |
| **Musculoskeletal disorders** |  |  |  |  |  |  |
| MSKD | parents | 16(612) | 16(900) | 0.019(0.005, 0.033) | 0.010 | 43.6% |
| **Genitourinary system disorders** |  |  |  |  |  |  |
| Pelvic inflammatory disease | parents | 10(1340) | 10(1210) | 0.121(0.096, 0.146) | <0.001 | 25.5% |
| **Conditions originating in the perinatal period** |  |  |  |  |  |  |
| ELBW/EP | parents | 4(1056) | 4(1100) | 0.075(0.048, 0.103) | <0.001 | 45.8% |
| VLBW/VP | parents | 6(1053) | 6(1053) | 0.009(-0.016, 0.034) | 0.481 | 98.9% |
| **Congenital malformations** |  |  |  |  |  |  |
| Spina bifida | caregivers | 2(125) | 2(132) | -0.044(-0.078, -0.010) | 0.012 | 93.2% |
| **Injury, poisoning and other consequences of external causes** |  |  |  |  |  |  |
| Injury | parents | 2(44) | 2(44) | -0.083(-0.155, -0.012) | 0.023 | 0.0% |
| **Chronic illness** |  |  |  |  |  |  |
| Chronic illness | parents | 6(164) | 6(164) | -0.047(-0.080, -0.013) | 0.007 | 45.8% |
| ODD: Oppositional defiant disorder; CD: Conduct disorder; DBD: Disruptive behaviour disorder; ADHD: Attention deficit hyperactivity disorder; SA: Substance abuse; VLBW/VP: Very low birth weight/ Very preterm; ELBW/EP: Extremely low birth weight/ Extremely preterm  *Health conditions were reported based on ICD-10 categories.  †Proxy-reported health utilities minus self-reported health utilities.  ‡Plus indicates patients have multiple diagnoses. | | | | | | |
